# The perspective of private practitioners regarding tuberculosis case detection and treatment delay in Amhara Region, Ethiopia: a cross-sectional study

**DOI:** 10.1186/1756-0500-4-285

**Published:** 2011-08-11

**Authors:** Solomon A Yimer, Carol-Holm Hansen, Gunnar A Bjune

**Affiliations:** 1 Norwegian Institute of Public Health, Postbox 4404, Nydalen 0403, Oslo, Norway; 2 Institute of Health and Society, Section for International Health, Faculty of Medicine, University of Oslo, Norway; 3 Amhara Regional State Health Bureau, P.O.Box 495, Bahir Dar, Ethiopia

**Keywords:** tuberculosis, private practitioners, treatment delay, Ethiopia

## Abstract

**Background:**

Engaging all health care providers in tuberculosis (TB) control has been incorporated as an essential component of World Health Organization's Stop TB Strategy and the Stop TB Partnership's global plan 2006-2015. Ethiopia has a growing private health sector. The objective of the present study was to investigate the role of private practitioners (PPs) in TB case detection and assess their perspectives on TB treatment delay in Amhara Region, Ethiopia.

**Results:**

A cross-sectional study among 112 PPs selected from private health facilities (PHF) in the region was conducted. The study was carried out between May and August 2008 and data was collected using a semi-structured questionnaire. Group differences were analyzed using one-way Anova test and a p-value of < 0.05 was considered statistically significant.

In this study, PPs saw a median of 12 TB suspects and 1.5 patients a week. The mean number of TB suspects and patients seen varied significantly among the different professions with p < 0.009 and p < 0.004, respectively. Pulmonary TB patients referred by PPs were delayed up to one week before starting treatment at government health facilities. A 22% increase in the detection of smear-positive TB cases may be achieved by involving all PHFs in the TB control program in the region. Nineteen percent of the PPs indicated that TB patients' prior attendance to non medical health providers resulted in complication of disease and increased treatment delay for TB.

**Conclusion:**

PPs manage a substantial number of TB suspects and patients in Amhara Region, Ethiopia. The GHF delay observed among TB patients referred by PPs to GHF is unnecessary. Expanding PPM-DOTS in the region and improving the quality of TB care at both government and private health facilities reduces treatment delay and increases TB case detection.

## Background

In most resource-poor countries the government has had the sole responsibility for the prevention and control of diseases of public health importance such as tuberculosis (TB) [1]. However, health services in general are rendered by both government and private health sectors. Recently, the World Health Organization (WHO) recognized that the global target of detecting 70% of all new TB cases is not likely to be met unless current efforts are strengthened by including important stakeholders in TB control [2]. Engaging all health care providers in TB control has thus been incorporated as an essential component of WHO's new Stop TB Strategy and the Stop TB Partnership's global plan 2006-2015 [2].

Private practitioners' (PPs) involvement in TB control has shown significant results in terms of case detection [1,3]. For example, several studies from India have demonstrated an increase in case detection ranging from 10% to 60% in private public mix (PPM) projects [4]. In addition, PPM substantially reduced the financial burden on patients and facilitated access to quality TB care [5].

TB case detection is very low throughout Ethiopia [6]. In 2008, the national smear-positive case detection rate (CDR) was estimated at 28%. In Amhara Region, the CDR was estimated at 21.5% in the same year [7] indicating an urgent need to utilize all the available opportunities in order to increase TB case detection and meet the 70% target as set by WHO.

Ethiopia has a growing private health sector. For many years, only government health facilities (GHFs) practiced the directly observed treatment short course (DOTS) strategy. Since 2007, selected private health facilities (PHFs) have been involved in the PPM-DOTS initiative [7]. However, despite the increasing number of PHFs, there is only one study from urban Ethiopia that described the number of TB patients managed by PPs [8]. Another study from Ethiopia indicated that treatment was delayed for TB patients who first visited PHFs [9]. The possible reasons for this delay have not been explored. Understanding the contribution of PPs in TB control is necessary to make recommendations that will reduce treatment delay for TB patients. The aim of this study was to investigate the role of PPs in TB case detection and assess their perspectives on TB treatment delay in Amhara Region, Ethiopia.

## Methods

### Setting

This study was conducted in Amhara Regional State which is the second largest region of Ethiopia. The region is divided into 11 zonal administrations. During the study period, there were 3 hospitals, 22 higher and 119 medium level clinics owned by the private sector in the region [7]. Hospitals and higher level clinics are managed by medical specialists and general medical practitioners (GPs) while medium level clinics are managed by GPs or health officers (HOs). Since 2008, 35 PHFs have been involved in PPM-DOTS in the region [7,10]. At the time of this study, the region also had 17 hospitals, 674 health centers and 2750 health posts owned by the government [7].

### Study design

A cross-sectional study was conducted between May and August, 2008. All PHFs with TB diagnostic facilities in Amhara Region were eligible for the study. Most PHFs had one PP who was the owner and the responsible health worker for the diagnosis and treatment of TB. But in the major towns of the region, there were 1-3 PPs/PHFs involved in the diagnosis and treatment of TB. From each PHF, one participant was enrolled in the study. Random sampling was applied to select the study participants in places where more than one PP/PHF was found.

### Definition of variables

**Figure 1:**
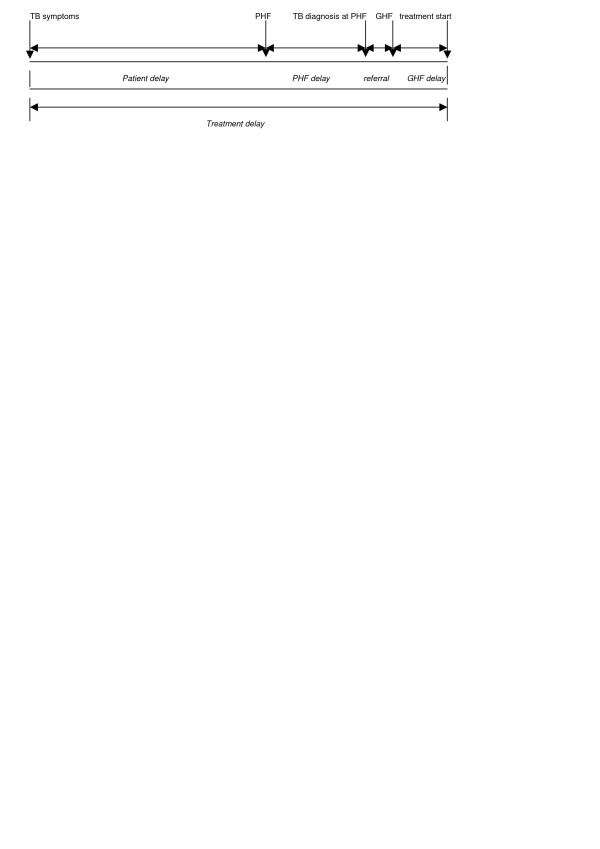
**Treatment delay: the delay period from the start of symptoms of TB until first start of treatment**. Private health facility (PHF) delay: the delay period from first visit of a TB patient to a PHF until first diagnosis of TB is made at PHF. Government health facility (GHF) delay: the delay period from first time a TB patient referred from PHF reported to GHF until treatment is initiated at GHF.

Medical providers- are health centers and hospitals with diagnostic and treatment facilities for TB. The relations between PHF, GHF and treatment delay are shown in Figure 1.

Medical specialists- are medical doctors with an MD degree plus a specialty certificate in internal medicine or pediatrics.

### Data collection and analysis

A semi-structured questionnaire was used to collect data. The questionnaire was pretested and the interviewers received adequate training before data collection commenced. Five health officers collected the data. The questionnaire addressed socio-demographic characteristics, number of TB suspects and patients managed by PPs and their perspectives on TB treatment delays. The interviewees were medical specialists, GPs and HOs.

Data was analyzed using SPSS version 16. After data entry and cleaning, proportions were computed and group differences were analyzed using one-way Anova tests. A p-value of < 0.05 was considered statistically significant.

We also conducted reviews of annual Tuberculosis and Leprosy Control Program (TBLCP) report, TBLCP guidelines [11] and held in-depth interviews with the TBLCP team leader and TBLCP experts at the regional level.

The study was approved by the Regional Committee for Medical Research Ethics in Eastern Norway (REK Øst), and the Ethiopian Science and Technology Agency in Addis Ababa, Ethiopia. Informed consent was taken from all study participants before the study was conducted.

## Results

### Characteristics of study participants

Among 144 PPs eligible for the study, a total of 112 (77.7%) PPs participated in the study. The vast majority, 110 (98.2%), were males. The mean age was 37.8 years. The median years of medical practice was 7.5 years (inter quartile-range [IQR] [3,10] (Table 1).

**Table 1:** Socio-demographic characteristics of the study participants

Variable	n (%)
**Sex**	
Male	110 (98.2)
Female	2 (1.8)
**Age**	
25-34	34 (30.4)
35-44	54 (48.2)
> 45	24 (21.4)
**Profession**	
Medical specialists	18 (16.1)
GPs	50 (44.6)
HO	44 (39.3)
**Medical practice**	
1-5 years	54 (48.2)
6-10 years	38 (33.9)
> 10 Years	20 (17.9)

Medical specialists: are medical doctors with an MD degree plus a specialty certificate in internal medicine or pediatrics.

GPs: General practitioners are practitioners with a first degree in medicine. They are authorized to provide clinical services to patients presenting at hospitals and higher or mid-level clinics.

HO: Health Officers are health professionals with a first degree in public health. They have adequate clinical training and are authorized to provide clinical services to patients presenting at mid-level clinics.

### Case detection

In this study, PPs had seen a significant number of smear-positive TB patients and suspects in their respective facilities. The median number of smear-positive cases detected per week was 1.5 (IQR 1, 2). Eighty four (75%) of the respondents diagnosed 1-3 smear-positive cases per week (Table 2). The median number of TB suspects detected per week was 12 (IQR 8, 20). The mean number of TB suspects and smear-positive cases identified varied significantly between the different PPs' professions with p < 0.009 and p < 0.004, respectively (Table 3).

**Table 2:** Number of TB suspects and smear-positive cases seen by PPs per week

Number of cases seen	n (%)
TB suspect	
1-10	49 (43.8)
11-20	39 (34.8)
> 20	24 (21.4)
Smear-positive TB cases	
0	16 (14.3)
1-3	84 (75.0)
> 3	12 (10.7)

**Table 3:** Analyses of mean difference for the number of TB suspects and smear-positive TB cases identified by the different professions of PPs

Profession	n (%)	TB cases mean (95% CI)	P-value	n (%)	TB suspects mean (95% CI)	p-value
Medical Specialists	18 (16.1)	3.3 (1.9, 4.7)	0.004	18 (16.1)	24 (14.1, 34)	0.009
GP	50 (44.6)	2.2 (1.4, 3.1)		50 (44.6)	16 (12.8, 22.1)	
HO	44 (39.3)	1.1 (.9, 1.3)		44 (39.3)	12 (10.1, 14.0)	

	112			112		

CI: confidence interval

### Perspectives on treatment delays

Patient's delay, i.e. the period from the onset of TB symptoms until the first visit to a medical provider, was estimated at 2-4 months by 88 (78%) of the PPs. Eleven (10%) of the PPs estimated patient's delay to be less than a month and the remaining 13 (12%) estimated patient's delay ranging from 4 months to years. The major reasons for patient's delay were perceived to be due to low awareness of TB among patients, lower/no access to medical services, self treatment and poverty.

The PHF delay was estimated within the range of 2 days to 15 days for smear-positive pulmonary TB (PTB) patients by 79% of the study participants, and the rest 21% of the respondents estimated a PHF delay ranging from 16 days to 21 days. PHF delay was also estimated for smear-negative PTB patients among eighteen PPs that had smear microscopy and x-ray facilities for TB. Accordingly, 12 (67%) of the PPs estimated a PHF delay of 15 days to 30 days for smear-negative PTB patients. The remaining 6 PPs (33%) estimated PHF delay to be more than 30 days. The reasons for the PHF delays greater than 30 days were perceived to be due to missed appointment dates by patients, incorrect diagnosis, lack/shortage of laboratory facilities and absence of laboratory technicians.

According to 77 (69%) of the respondents, TB patients delayed after they were referred from PHFs to the GHF for treatment. The mandatory repeat smear microscopy exam policy at the GHFs was the major reason for 2-3 days delay before starting treatment. The condition was worse for patients who were referred on Fridays. Since Saturday and Sunday are not regular working days, patients had to wait until Monday for the repeat smear microscopy at the GHF. According to the respondents, these patients could experience a delay of up to one week after referral before starting treatment. Collaboration with government

Most of the PPs reported that their working relationship with the government has improved. The recent progress made in terms of commencing PPM-DOTS in selected PHFs in the region has been considered a positive step towards strengthening good collaboration. PPs were also asked about the types of measures needed to reduce TB treatment delay in the region. The majority stated that the government health workers must respect the PPs professionally, and almost all suggested that PHFs should be fully authorized to manage TB patients. In addition, uninterrupted supply of diagnostic reagents and supplies, different recording and reporting formats, and establishing a coordinating committee including private and government representatives were important interventions needed to strengthen collaboration and provide quality care to TB patients.

In this study, 19 (17%) of the respondents expressed a concern over the growing involvement of non medical providers in TB care. According to these respondents, a number of TB suspects and patients visited non medical providers before they reported to a medical facility for their symptoms. In addition, many patients on anti-TB regimens were being advised by non medical providers to stop their medication and start herbal or other traditional remedies. Most of the patients followed the advice of the non medical providers, their conditions worsened and thereafter reported to the PHFs for better management.

## Discussion

Following the WHO-recommended strategy of engaging all health providers in TB care, PPM-DOTS initiatives have been expanded as an essential tool of improving TB case detection in many high TB burden countries. In this study, the role of PPs with respect to TB case detection was investigated. The results showed that the median number of TB suspects and patients seen was high indicating that PPs were potential sources of health care for TB patients in the study region. Our finding is higher than that reported in Indonesia [12] and Pakistan [13] but lower than that in the Philippines [14].

The mean number of TB suspects and patients seen by PPs varied according to profession of the practitioner. More cases were seen by specialist doctors. The reason could be that medical specialists in the study region work in hospitals and higher level clinics where the patient load is high. In another study, medical specialists were also found to have seen more cases compared to other PPs [12].

Previous studies have shown that TB patients attending PHFs were significantly delayed before starting treatment [9,15-17]. In our study, TB patients referred from PHFs to GHFs were reported to have been delayed up to one week before starting treatment. The main reason for the delay was a result of the mandatory repeat sputum smear microscopy exam policy at the GHF. This has serious implications for TB control. The delay caused by mandatory repeat examinations may worsen the patient's illness, increase the risk of further transmission and incur extra costs for the patient. We therefore suggest that the government should look into the possibility of providing routine health service for TB patients during weekends so that those referred from PPs receive timely diagnosis and treatment service. This may reduce GHF delay and extra cost incurred by patients due to the unnecessary delay.

The PHF delay was estimated within the range of 2 days to 15 days for smear-positive PTB patients. This is contradictory to the findings of a previous study [9]. Given the long course of TB disease progression and other factors such as the initial prescription of broad spectrum antibiotics for TB suspects, it is likely that the PHF delay in our study was underestimated. Also, when we look at the PPs' perception about the magnitude of and factors for patient's delay, a majority of the PPs perceived that the patient's delay would be estimated at 2-4 months. The major factor for the delay was perceived as lack of knowledge of TB among patients and may generally imply blame to the patient. In contrast, many delay studies that focused on TB patients' perspectives have indicated that the major factor for treatment delay is a "vicious cycle" of repeated visits to the same level of health care suggesting that medical providers are to blame for the delay [18,19]. We recommend that a forum should be organized to discuss the available evidence on treatment delay in TB among private and government health workers.

In this study, a considerable proportion of PPs indicated that many TB patients visited non medical health providers before reporting to PHFs. According to WHO, involving all health providers in TB control is one of the strategies that will increase TB case detection [2]. However, non medical health providers in this study were perceived to have participated in a manner that compromised the TB control effort. This is serious as it may contribute to the transmission of drug resistant TB in the region. A mechanism should be devised to involve non medical providers at an appropriate level and with a clearly defined role in TB control efforts. Additional studies investigating the role of non medical providers in the management of TB in the region are warranted.

Our review of the Amhara Regional Health Bureau report showed that 35 PHFs were participating in PPM-DOTS [7]. Taking all PHFs with TB diagnostic facilities in the region into account, the participation of PHFs in PPM-DOTS was 24%. This indicates that the involvement of PHFs in PPM-DOTS in the study region was low.

Our results indicate that a median number of 12 TB suspects were seen per week. All PPs in the region may thus see a total of 72,576 TB suspects in one year. In 2 previous studies, the prevalence of smear-positive TB in urban and rural Ethiopia was estimated at 13% [20] and 3.8% [21], respectively. By using a mean prevalence of 8.4% from the two studies as a smear-positive TB prevalence estimate for the study region, a total of 6,096 smear-positive cases may be detected among the expected 72,576 TB suspects in the region. The study region has a population of 17.2 million [22], and a smear-positive TB incidence of 163/100,000 population [6]. This means that a total of 28,036 new cases may be expected per year. Hence, taking the above information into our calculation, the region may achieve a 22% increase in the detection of smear-positive TB cases by involving all PHFs in PPM-DOTS.

This study has some limitations. PPs were asked retrospectively to state the number of TB suspects and patients seen in their practice. This data may be influenced by recall bias. A prospective recording and observation would have provided an indication as to whether or not the PPs' reported estimates genuinely reflected the actual number of patients and suspects seen. Such a study was not within the scope of our limited resources. However, the PPs were asked to state the number of cases seen per week rather than per month or year in an effort to shorten the time gap and obtain the most correct recall data.

The study may also be subject to selection bias. Among all PHFs eligible for the study, 32 (22%) of them did not participate mainly because we could not find them despite our visits. However, even if we were not able to include all PHFs eligible for the study, majority of the PHFs that were actively rendering service to the population in the region were included from all administrative levels (zones and districts) in the region. Thus, we believe that the possibility of selection bias in this study is reduced.

## Conclusions

Our findings indicate that there is a huge potential for TB control in Amhara Region, Ethiopia. PPs see a large number of TB suspects and patients in the region. The GHF delay observed among TB patients referred by PPs to GHF is unnecessary, and is one of the reasons for increased treatment delay among TB patients seeking initial medical attention from PPs. More PHFs should thus be involved in PPM-DOTS in order to reduce treatment delay and increase case detection of TB in the region.

## Abbreviations

CDR: case detection rate; DOTS: directly observed treatment short course; GHFs: government health facilities; GPs: general medical practitioners; HOs: health officers; IQR: inter quartile-range; PHFs: private health facilities; PPs: private practitioners; PTB: pulmonary tuberculosis; REK Øst: Regional Committee for Medical Research Ethics in Eastern Norway; TB: tuberculosis; TBLCP: Tuberculosis and Leprosy Control Program; PPM: private public mix; WHO: World Health Organization.

## Competing interests

The authors declare that they have no competing interests.

## Authors' contributions

SAY conducted the data collection. SAY, GAB and CHH performed the data analysis. SAY drafted the manuscript. The three authors edited and approved the final manuscript.
